# Serum interleukin-6 level and its association with pulmonary involvement in progressive systemic sclerosis; a case-control study 

**DOI:** 10.1186/s12948-023-00188-1

**Published:** 2023-09-05

**Authors:** Ahmad Piroozmand, Batool Zamani, Hamed Haddad Kashani, Javad Amini Mahabadi

**Affiliations:** 1 Autoimmune Diseases Research Center, Kashan University of Medical Sciences, Kashan, Iran; 2 Anatomical Sciences Research Center, Institute for Basic Sciences, Kashan University of Medical Sciences, Kashan, Iran; 3 Gametogenesis Research Center, Institute for Basic Sciences, Kashan University of Medical Science, Kashan, Iran

**Keywords:** Systemic sclerosis, Pulmonary Involvement, Interleukin-6, EUSTAR score

## Abstract

**Background:**

Primary Systemic Sclerosis (PSS) is a connective tissue disorder characterized by excessive collagen deposition in the skin and internal organs. Interstitial lung disease (ILD) is a late demonstration of PSS and cytokines can contribute to the disease pathology. The purpose of the current study was to determine the association between serum interleukin-6 level and pulmonary involvement in progressive systemic sclerosis.

**Methods and materials:**

Demographic data and serum interleukin-6 levels were measured for 30 PSS patients with pulmonary involvement (case group) and 30 PSS patients without pulmonary involvement (control group) following informed consent. The disease duration and activity, C-reactive protein (CRP), chest x-ray and highresolution CT scan (HRCT) findings, ejection fraction (EF) and echocardiography findings, and pulmonary artery pressure (PAP) were also determined in both groups.

**Results:**

The age of patients in case and control groups was 52.5 ± 9.3 and 43.9 ± 9.7 years, respectively (p = 0.001). No significant difference was found between serum levels of IL-6 in case and control groups (73.1 ± 95.4 vs 46.7 ± 83.6 pg/ml, p = 0.267). However, IL-6 level was significantly higher in male case patients compared to male controls (p = 0.007). The duration of PSS was 11.6 ± 6.4 and 7.4 ± 4.2 years in case and control groups, respectively (p = 0.002). The quantitative CRP and PAP was also significantly higher in case patients (p = 0.01 and p < 0.001, respectively). There was found reticulonodular pattern in 20 (66.7%) of the cases, whereas 28 (93.3%) of the controls had normal Chest X-rays (CXR) (p < 0.001). EF was significantly lower in case patients compared to control patients (p = 0.001).

**Conclusion:**

The serum level of IL-6 did not appear to have a relationship with pulmonary involvement, hence it could not be regarded as a potential therapeutic target.

## Introduction

Primary systemic sclerosis (PSS) is a systemic collagen vascular disease of unknown origin, which is characterized by the involvement of vessels and connective tissue. It is remarkably associated with interstitial lung disease (ILD) with a prevalence of 25–65%, which is higher than that of other collagen vascular diseases [1–3]. ILD in PSS patients is characterized by either alveolitis or fibrosis [4]. Alveolitis is the inflammation of lung tissue in the initial stages of ILD, which is gradually aggravated by fibrosis and ends in restrictive lung disease. One of the most important causes of mortality and morbidity among PSS patients is lung disease [4]. Alveolitis is amenable to treatment in early stages, but once leading to fibrosis, it would be irreversible and will cause severe morbidity in PSS patients [5]. The diagnosis of lung disease in these patients entails a number of diagnostic tests such as chest X-ray, spirometry, High-Resolution CT Scan (HRCT), bronchoscopy, and lung fluid aspiration and flow cytometry, which are expensive and time-consuming. Moreover, during the early stages, chest X-rays might be normal and more advanced diagnostic tests are required.

Although the exact pathogenesis of scleroderma is unknown, a couple of cells and cytokines are known to be involved in the process of fibrosis in this disease, e.g. Interleukin 6 (IL-6) released by inflammatory cells [6]. Previous studies in PSS patients demonstrated the rise of IL-6 serum level and its positive relationship with the severity of skin involvement [7, 8]. IL-6 is an inflammatory cytokine released from T-helper 2 (Th2) cells, which is a therapeutic target in PSS patients. The hypoxia caused by vasculopathy, which plays a major role in the pathogenesis of scleroderma, affects the transcription of IL-6 gene in PSS [6]. Moreover, it was shown in recent in vitro studies that it is involved in the activation and apoptosis of endothelial cells [9].

Due to the high prevalence of lung disease in PSS patients, hence the high morbidity, and that some of the diagnostic tests are invasive and costly, there is a clear need for a less costly and invasive diagnostic technique with an acceptable level of sensitivity and specificity to diagnose the lung disease in an earlier stage and prevent the irreversible complications. Therefore, this study was done to study the role of IL-6 in the pathogenesis of lung disease in progressive systemic sclerosis in patients diagnosed with PSS in the rheumatology clinic of Kashan Shahid-Beheshti hospital over 2015–2016, so as to be helpful in the early diagnosis and follow-up of treatment and complications in PSS patients.

## Methods and materials

In this case–control study [10], 60 patients diagnosed with PSS which received medical care in the rheumatology clinic of Kashan Shahid-Beheshti hospital over 2015–2016 were studied. All the patients were diagnosed by an expert rheumatologist based on American College of Rheumatology (ACR) criteria for PSS. The patients were studied in two groups of case (N = 30) which had ILD as confirmed by the radiologist in their chest X-ray and lung HRCT, and control (N = 30) which had no pulmonary involvement in radiologic studies. The patients were enrolled in the study only after giving informed consent. To determine the activity of systemic sclerosis, the EUSTAR scoring scale was used. The demographic data including age, sex, and disease duration was obtained by taking history of patients and was recorded in the designed patient forms [11].

### The measurement of IL-6 serum level

A blood sample of five milliliters from each patient was centrifuged with 1300 rpm for 10 min, and the supernatant solution was then frozen in − 20 °C until the completion of sampling from the patients. All the frozen samples were then left to reach laboratory temperature and were subsequently used for ELISA test. The Diacolon kit made by France, which was purchased form Padginteb Co. and had a kit sensitivity of 2 pg/mL was used for ELISA test. To measure the sample concentration of IL-6, 50 µL of Streptavidin-HRP was added to each well. Then, 40 µL of prepared serum samples was added to each well, followed by 10 µL of antibody and 50 µL of Streptavidin-HRP. The wells were incubated in 37 °C temperature for 60 min by shaking method and thereafter were diluted 30 times by instilled water and underwent washing. Then, 50 µL of chromogen solution A and 50 µL of chromogen solution B were added to each well and the wells were incubated out of light in 37 °C temperature for 10 min. Finally, 50 µL of Stop Solution was added to each well, and IL-6 serum concentration was obtained according to the standard concentration and corresponding optical density (OD) values.

### Data analysis and statistical method

The crude data was analyzed using SPSS-16 [12, 13]. The measures of central tendency like mean, frequency, and standard deviation were calculated [14, 15]. The data was described using descriptive statistics and was analyzed by Chi-squared and Fischer’s exact tests [16, 17]. To compare the IL-6 level of the two groups of PSS patients with and without lung involvement, Mann–Whitney test was used. The significance level was assumed p-value of less than 0.05 [18, 19].

## Results

This study was done on the PSS patients receiving medical care in the rheumatology clinic of Kashan Shahid-Beheshti hospital over 2015 to 2016 and it was aimed to study the relationship between the serum level of IL-6 and the involvement of lung as ILD in PSS patients. The patients were studied in two groups of PSS with ILD as cases (N = 30) and those without ILD as controls (N = 30). All the patients of case group had diffuse PSS, while 85% of the patients of control group had the diffuse type. In the case group, there were 22 females (73.4%) and 8 males (26.6%), while in the control group there were 18 females (60.0%) and 12 males (40.0%) (Table 1). The average age was 52.5 ± 9.3 years in the case group, and 43.9 ± 9.7 in the control group, the difference of which was significant (p = 0.001) (Table 1). The disease duration was 11.6 ± 6.4 years in the case group, and 7.4 ± 4.2 in the control group, which showed significant difference (p = 0.002) (Table 1). As it is shown in Table 1, the frequency of scleroderma activity based on EUSTAR score was significantly different between the two groups (p < 0.001). The mean serum levels of IL-6 and hemoglobin of the two groups are shown in Table 1. The serum levels of IL-6 were 73.1 ± 95.4 and 46.6 ± 83.6 pg/mL in case and control groups, respectively, the difference of which was not significant (p = 0.267).

**Table 1:** Demographic data, frequency of scleroderma activity based on EUSTAR score and serum Levels of IL-6 and hemoglobin in both groups of the study

Variable	Case group (N = 30)	Control group (N = 30)	P-value
Age (years)	52.5 ± 9.3	43.9 ± 9.7	0.001^a^
Sex			
Male	8 (26.6%)	12 (40.0%)	0.237^b^
Female	22 (73.4%)	18 (60.0%)	
Disease duration (years)	11.6 ± 6.4	7.4 ± 4.2	0.002^a^
Scleroderma activity (EUSTAR score)			
Active	21 (70%)	6 (20%)	< 0.001
Inactive	9 (30%)	24 (80%)	
Serum level of IL-6 (pg/mL)	73.1 ± 95.4	46.7 ± 83.6	0.267
Hemoglobin (gr/dL)	12.5 ± 1.4	13.0 ± 1.7	0.257

All data was shown into Mean ± Standard Deviation

^a^Mann-Whitney test

^b^Chi-square test

As seen in Table 2, the mean serum level of IL-6 in males was 141.7 ± 103.2 and 14.3 ± 19.3 pg/mL in the case and control groups, respectively, the difference of which was significant (p = 0.007). However, it was 48.1 ± 81.2 and 68.2 ± 102.2 pg/mL in females of the case and control groups, respectively, which showed no significant difference (p = 0.693). As evident in Table 2, the mean serum level of IL-6 in those with active disease was 79.9 ± 94.3 and 51.1 ± 88.1 pg/mL in the case and control groups, respectively, the difference of which was not significant (p = 0.414). Moreover, in those with inactive disease, it was 57.1 ± 101.8 and 45.6 ± 84.4 pg/mL in the case and control groups, respectively, which showed no significant difference as well (p = 0.793). The mean serum levels of IL-6 in terms of age are shown in Table 2. In patients less than 50 years old, it was 32.7 ± 59.1and 55.5 ± 93.3 pg/mL in the case and control groups, respectively, the difference of which was not significant (p = 0.987). Furthermore, in those aged 50 years or more, it was 103.9 ± 107.5 and 17.6 ± 23.9 pg/mL in the case and control groups, respectively, which showed no significant difference as well (p = 0.057). As seen in Table 2, the mean serum level of IL-6 in those with a disease duration less than 10 years was 103.9 ± 110.0 and 51.4 ± 92.6 pg/mL in the case and control groups, respectively, the difference of which was not significant (p = 0.140). Moreover, in those with a disease duration of 10 years or more, it was 59.8 ± 88.0 and 27.6 ± 22.2 pg/mL in the case and control groups, respectively, which showed no significant difference as well (p = 0.770).

**Table 2:** The mean serum level of IL-6 (pg/mL) in terms of sex, age and disease duration in both groups of the study

Parameters	Group	Frequency	Mean ± Standard deviation	P-value^a^
Sex				
Male	Case	8	141.7 ± 103.2	0.007
	Control	12	14.3 ± 19.3	
Female	Case	22	48.1 ± 81.2	0.693
	Control	18	68.2 ± 102.2	
Active	Case	21	79.9 ± 94.3	0.414
	Control	6	51.1 ± 88.1	
Inactive	Case	9	57.1 ± 101.8	0.793
	Control	24	45.6 ± 84.4	
Age				
< 50 years	Case	13	32.7 ± 59.1	0.987
	Control	23	55.5 ± 93.3	
≥ 50 years	Case	17	103.9 ± 107.5	0.057
	Control	7	17.6 ± 23.9	
Disease duration				
< 10 years	Case	9	103.9 ± 110.0	0.140
	Control	24	51.4 ± 92.6	
≥ 10 years	Case	21	59.8 ± 88.0	0.770
	Control	6	27.6 ± 22.2	

All data was shown into Mean ± Standard Deviation

^a^Mann-Whitney test

In Table 3, the frequencies of the involved organs by PSS and concurrent other rheumatologic diseases are shown for both groups of the study. As it can be seen, eight patients (26.6%) had cardiac involvement in the case group. There were also five patients (16.7%) with concurrent other rheumatologic diseases in the case group. The results of qualitative CRP test with the help of Bionic and Omega kits were reported as negative, one positive, two positive and three positive. In the past, most CRP levels were reported qualitatively or semi-quantitatively. But today, more accurate and sensitive immunoturbidometry, ELISA and luminescence methods have been developed to measure this parameter. Considering that it is based on passive agglutination, which is relatively sensitive, that is why the report was presented as negative, + 1, + 2 and + 3 or regional phenomenon. Therefore, it is suggested to use quantitative methods in future research. As evident in Table 3, (26.7%), 9 (30%), 7 (23.3%), and 6 (20%) patients in the case group, and 17 (56.7%), 5 (16.7%), and 1 (3.3%) patients in the control group had a qualitative CRP of Negative, + 1, + 2, and + 3, respectively. The difference between the two groups was significant (p < 0.05).

**Table 3:** The frequencies of the involved organs by PSS, concurrent other rheumatologic diseases and qualitative CRP in both groups of the study

Variable	Case group (N = 30)	Control group (N = 30)	P-value
Skin involvement	30 (100%)	30 (100%)	1^a^
Cardiac involvement	8 (26.6%)	0 (0%)	0.003^a^
Heart failure	1 (12.5%)	0 (0%)	
Pericarditis	7 (87.5%)	0 (0%)	
Other rheumatologic diseases	5 (16.7%)	6 (20%)	0.739^a^
Osteoporosis	5 (100%)	2 (33.3%)	
Rheumatoid arthritis	0 (0%)	2 (33.3%)	
Polymyositis	0 (0%)	1 (16.7%)	
Sjögren disease	0 (0%)	1 (16.7%)	
Qualitative CRP			
Negative	8 (26.7%)	17 (56.7%)	0.010^a^
+ 1	9 (30%)	7 (23.3%)	
+ 2	7 (23.3%)	5 (16.7%)	
+ 3	6 (20%)	1 (3.3%)	

All data was shown into number (percent)

^a^Fisher’s exact test

The frequency distribution of chest X-ray findings is shown in Table 4. The most common finding was reticulonodular pattern (66.7%) in the case group, and normal pattern (93.3%) in the control group with the difference being significant (p < 0.001). The lung HRCT finding of the greatest frequency was honeycombing (76.7%) in the case group, while all the patients of the control group had normal pattern with the difference being significant (Table 4). As shown in Table 4, 26 patients (86.7%) in the case group and one patient (3.3%) in the control group demonstrated restrictive respiratory pattern, the difference of which was significant (p < 0.001). As evident in Table 4, there were 10 patients (33.3%) with abnormal echocardiography pattern in the case group, whereas all the patients of the control group had normal echocardiography, the difference of which was significant (p = 0.001). The heart ejection fraction (EF) of the greatest frequency was 55% (11 patients) in the case group and 60% (17 patients) in the control group, the difference of which was significant (Table 4). As shown in Table 4, the most frequent pulmonary artery pressures (PAP) were 45 and 50 mmHg (six and six patients, respectively) in the case group, while it was normal (25 patients) in the control group with the difference being significant (p < 0.001).

**Table 4:** The frequency distribution of chest X-ray, HRCT, Spirometry Pattern, Echocardiography findings, ejection fraction and pulmonary artery pressure in both groups of the study

Variable	Case group	Control group	P-value^a^
Chest X-ray finding			
Normal	1 (3.3%)	28 (93.3%)	> 0.001
Reticulonodular pattern	20 (66.7%)	2 (6.7%)	
Fibrotic pattern	9 (30%)	0 (0%)	
Total	30 (100%)	30 (100%)	
HRCT finding			
Normal	0 (0%)	30 (93.3%)	> 0.001
Honeycombing	23 (76.7%)	0 (0%)	
Fibrosis	5 (16.7%)	0 (0%)	
Peribronchial thickening	2 (6.7%)	0 (0%)	
Total	30 (100%)	30 (100%)	
Spirometry pattern			
Normal	4 (13.3%)	29 (96.7%)	> 0.001
Restrictive	26 (86.7%)	1 (3.3%)	
Total	30 (100%)	30 (100%)	
Echocardiography finding			
Normal	20 (66.7%)	30 (100%)	> 0.001
Abnormal	10 (33.3%)	0 (0%)	
Total	30 (100%)	30 (100%)	
Ejection fraction (%)			
40	1 (3.3%)	0 (0%)	> 0.001
45	3 (10%)	0 (0%)	
48	1 (3.3%)	0 (0%)	
50	5 (16.7%)	0 (0%)	
55	11 (36.7%)	12 (40%)	
60	8 (26.7%)	17 (56.7%)	
65	0 (0%)	1 (3.3%)	
67	1 (3.3%)	0 (0%)	
Total	30 (100%)	30 (100%)	
PAP (mmHg)			
Normal	4 (13.3%)	25 (83.3%)	> 0.001
20	5 (16.7%)	2 (6.7%)	
25	2 (6.7%)	1 (3.3%)	
40	2 (6.7%)	2 (6.7%)	
45	6 (20%)	0 (0%)	
50	6 (20%)	0 (0%)	
55	2 (6.7%)	0 (0%)	
60	2 (6.7%)	0 (0%)	
63	1 (3.3%)	0 (0%)	
Total	30 (100%)	30 (100%)	

All data was shown into number (percent)

*PAP* Pulmonary Artery Pressure

^a^Fisher’s exact test

## Discussion

To study the relationship between the serum level of IL-6 and lung involvement in the form of ILD in PSS, 30 PSS patients with ILD (cases) and 30 without ILD (controls) were studied. The patients received medical care in the rheumatology clinic of Kashan Shahid-Beheshti hospital over 2015–2016. The disease duration was 11.6 ± 6.4 years in the case group and 7.4 ± 4.2 in the control group, which showed a significant difference (p = 0.002). The mean serum level of IL-6 was 73.1 ± 95.4 and 46.7 ± 83.6 pg/mL in the case and control groups, respectively, the difference of which was not significant (p = 0.267). The mean serum level of IL-6 in males was 141.7 ± 103.2 and 14.3 ± 19.3 pg/mL in the case and control groups, respectively, the difference of which was significant (p = 0.007). It has been determined that IL-6 marker elevates in males in comparison to females [20]. The reason why males tend to develop hepatocellular carcinoma (HCC) more often than females, was studied elegantly in a diethylnitrosamine-induced mouse model of HCC and it was found that IL-6 mediated inflammation in Kupfer cells mediated via a co-adapter protein Myd 88, which is more frequently seen in males [21]. However, it showed no significant difference in females with serum levels of 48.1 ± 81.2 and 68.2 ± 102.2 pg/mL in the case and control groups, respectively (p = 0.693). The serum level of IL-6 demonstrated no significant difference between the two groups in terms of disease activity, age, and disease duration.

In a study by Michele Ludici et al. in 2015, it was demonstrated that ILD affects almost 90% of Systemic Sclerosis (SSc) patients and is associated with decrease in Forced Vital Capacity (FVC), pulmonary fibrosis in HRCT, increase in serum level of IL-6, detectable serum level of anti-topoisomerase antibody, and diffuse skin involvement, which is consistent with the results of our study [22].

In a study by Jurisic et al. in 2013 on 31 patients with PSS and 31 healthy people, the routine echocardiography showed normal left ventricular EF (LVEF), while pulsed-wave Doppler echocardiography proved low LVEF and high early diastolic velocity and E/e ratio in the patients. Despite normal routine echocardiography, it was demonstrated that there was a relationship between the myocardial dysfunction proved by pulsed-wave echocardiography and serum level of IL-6. Furthermore, there was found a relationship between disease activity (EUSTAR score) and left ventricular dysfunction and serum level of IL-6. In our study, the case group showed a lower EF than that of the control group, which is consistent with the result of Jurisic study. The disease activity based on EUSTAR score and disease duration in Jurisic study were found to have a relationship with serum level of IL-6, which is contrary to the results of our study [23].

In a cohort study by Lauretis et al. in 2013, the serum level of IL-6 was measured by ELISA technique in 212 patients with SSc-ILD. The mortality rate and the decrease in pulmonary function were also studied. It was shown that the cut-off serum level of IL-6 capable of predicting FVC and DLCO reduction over one year and mortality over 30 months was IL-6 > 7.67 pg/mL. After stratifying based on the severity of restrictive disease, serum level of IL-6 was only capable of predicting the aforementioned parameters in those with mild ILD. It was finally concluded that serum IL-6 level is a predictor of ILD aggravation only in patients with mild ILD [24]. Nevertheless, our study demonstrated no significant relationship between serum level of IL-6 and ILD.

In a study by Schmidt et al. in 2009, the cytokines in the bronchoalveolar lavage (BAL) fluid were studied. This study was done on 32 PSS patients with ILD and 26 healthy patients. The measurement of cytokine concentrations of BAL fluid, pulmonary function test, and HRCT were done. There was found a significant increase in the BAL fluid levels of IL-4, IL-6, IL-7, and IL-8, and a significant relationship with pulmonary fibrosis in the patients, hence the role of BAL fluid cytokines in ILD pathophysiology. In this study, there was a significant increase in IL-6 in those patients with neutrophilic alveolitis compared with the control group [25]. However, in our study, the IL-6 level was measured in serum, which showed no significant relationship with ILD.

In a Japanese study in 1996, to study the prognostic factors and mortality causes of systemic sclerosis, 496 Japanese PSS patients were followed for 5–20 years. The most frequent mortality causes were cardiac, pulmonary, and kidney failure, and pulmonary fibrosis. This highlights the importance of lung involvement screening during the early stages of the disease [26].

In another study by Crestani et al. in 1994, 11 PSS patients with lung involvement and eight healthy people were studied by measurement of IL-6 level in serum, BAL fluid, blood monocytes, and alveolar macrophages. It was shown that the serum and BAL fluid level of IL-6 was the same in both the case and control group, on the contrary to the result of our study, while the blood monocytes and alveolar macrophages of PSS patients with lung involvement secreted a higher concentration of IL-6 than those of the control group [27].

In a study by Yousif M et al. on patients with systemic sclerosis (SSc) and controls, the serum level of IL-6 was found to be significantly higher in SSc patients than that of controls. Nevertheless, in our study, the serum level of IL-6 was higher in PSS patients with ILD than that of PSS patients without ILD, the difference of which was not significant. However, the difference was found to be significant in male patients.

It was also found in our study that qualitative CRP was significantly higher in PSS patients with ILD than that of those patients without ILD, which is in line with the results of Yousif M et al. study [28].

## Conclusion

The serum level of IL-6 did not appear to have a relationship with pulmonary involvement, hence it could not be regarded as a potential therapeutic target. Since a difference of IL-6 levels was observed only in male with PSS, the correlations of the previously indicated clinical, instrumental and laboratory parameters with IL-6 levels of this group must be reported in the future researches.

## Data Availability

The dataset used in this study is available with the authors and can be made available upon request.
